# Isolated Non-Traumatic Bilateral Coronoid Process Fracture of the Mandible

**DOI:** 10.7759/cureus.829

**Published:** 2016-10-13

**Authors:** Nabil Al-Khalisi, Krishna Kumar, Natasha Acosta

**Affiliations:** 1 Department of Radiology, Truman Medical Center, University of Missouri School of Medicine, Kansas City, MO, USA

**Keywords:** mandible, coronoid process, bilateral fractures

## Abstract

Isolated bilateral fractures of the coronoid processes of the mandible occurred in this patient without any significant trauma. The definite etiology of this case is unknown, but possible causes or contributing factors may include acute reflex contraction of the patient’s temporalis muscles leading to bilateral stress fractures, coronoid process hyperplasia, or the patient’s long-term use of omeprazole. The planned treatment for this patient included pain control with Mobic and tramadol and splint fabrication followed by arch bar placement with training elastics for six weeks.

## Introduction

According to a previous study by Fridrich, et al. [1], in the United States, coronoid process fractures account for 1.3% of all mandibular fractures. In this same study of patients with mandibular fractures, 53% had unilateral fractures of the mandible, 37% had bilateral fractures of the mandible, and 43% of these patients had an associated injury (i.e. head injuries, head and neck lacerations, midface fractures, or cervical spine fractures). In a study done by Rapidis, et al. [2], it was reported that bilateral fractures of the coronoid process of the mandible were associated with a zygomatic arch or other facial injuries. Isolated coronoid fractures are very uncommon because the coronoid process is anatomically protected by the zygomatic arch and its associated muscles [3]. Most coronoid fractures are due to indirect blunt or penetrating trauma [4]. To our knowledge, only one case of bilateral isolated coronoid fractures has been reported in the literature [5] and it was reported with trauma. No cases of bilateral isolated coronoid fractures without trauma have been reported in the literature. Verbal informed consent was obtained from the patient for this study.

## Case presentation

A 36-year-old female presented to the oral and maxillofacial surgery (OMFS) clinic at Truman Medical Center (TMC), United States, stating that she sustained a left coronoid process fracture one year prior. The patient reported that she was driving in her vehicle and pulled into her driveway at home when suddenly she heard a pop and then felt facial pain, but repeatedly denied any trauma to her facial area. Her pain was associated with migraine-type headaches, otalgia, and malocclusion. Following this injury, the patient went to her primary care physician who obtained plain radiographs. The patient reported she has had “bad temporomandibular joint (TMJ) problems” since her childhood and has seen multiple specialists but to no relief. The patient had a history of maxillary orthodontic closure of a diastema in high school, which was removed after one year. The patient has had no orthodontics performed to the mandibular arch. Her medication regimen at this time included tramadol, Soma, oral contraceptives, omeprazole, diclofenac, spironolactone, and multivitamins. Omeprazole and diclofenac were discontinued at this visit and the patient was started on Mobic.

Upon examination, the patient had prognathism of her lower jaw with occlusion of only one tooth, which was comfortable for her. She had a maximal incisal opening of 35 mm. When asked to bite, the patient’s teeth touched on the left side only and demonstrated a right posterior open bite. Upon request to intercuspate her teeth, the patient was able to do so but had a transverse discrepancy with a crossbite on the right in the posterior aspect. She had lost her ability to find her own bite. The patient exhibited capsulitis, especially on the left side. The following muscles were tender to palpation: right and left temporalis, right and left masseter, sternocleidomastoid, and trapezius. Additionally, the bilateral shoulder girdles and bilateral scapula were tender to palpation.

**Figure 1:**
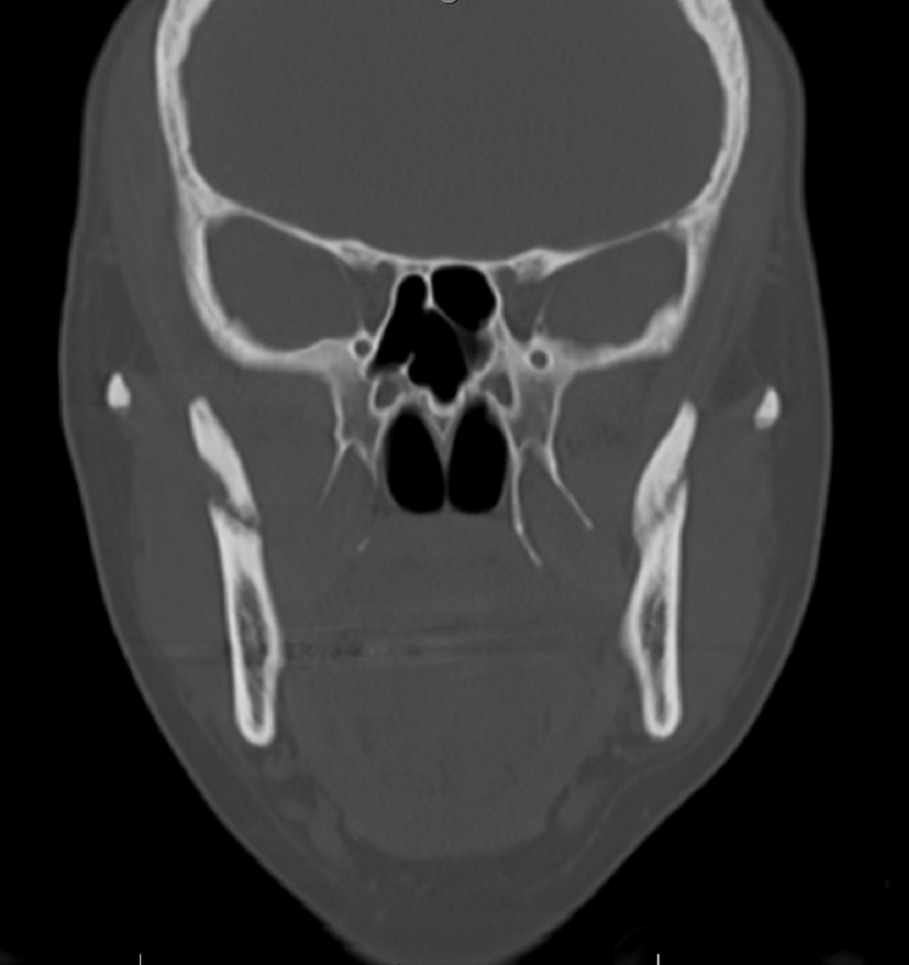
Coronal CT shows isolated bilateral coronoid process fracture

**Figure 2:**
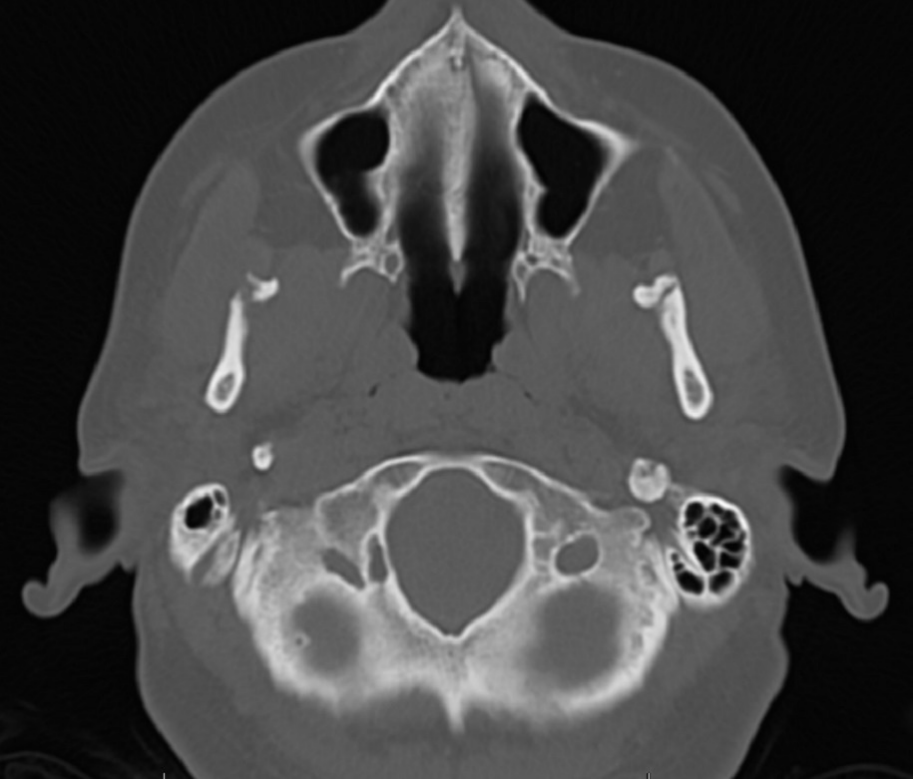
Axial CT shows isolated bilateral coronoid process fracture

The radiographic examination performed by panoramic X-rays of the mouth by her primary care physician one-year prior revealed visible bilateral coronoid fractures as well as a left zygomatic arch linear non-displaced fractures. A magnetic resonance imaging (MRI) of the right and left TMJs and a computed tomography (CT) scan of the face without contrast were ordered by the OMFS clinic at TMC. The MRI of the TMJs revealed bilateral coronoid process fractures without significant displacement, severe degenerative changes of the left TMJ with the partial erosion of the left mandibular eminence, and a large joint effusion of the left TMJ. The MRI also revealed the left TMJ disc was anteriorly displaced without reduction on closed and open mouth views. The CT of the face revealed bilateral subacute coronoid process fractures without significant displacement, with callus/healing changes greater on the left side compared to the right, and asymmetrically advanced left TMJ degenerative changes. Figures 1-2 show the CT images of our case in two planes.

The planned treatment for the patient included keeping the patient on a soft diet and splint fabrication to get the patient into a normal bite. Pain control was to be continued with tramadol, Mobic, and Soma. The splint fabrication was followed by open reduction and internal fixation (ORIF) surgery (surgical application of arch bars arthrocentesis with arthroscopy for maxillomandibular fixation and left TMJ manipulation, with training elastics for up to six weeks).

## Discussion

Isolated coronoid process fractures of the mandible are very rare because of their protected position under the zygomatic bone and its associated muscles. A fracture of the coronoid process mostly results from either direct/penetrating trauma to the bone or if there is a sudden contraction of the temporalis muscle at the time of impact [5]. However, in this patient there was no significant direct head trauma and no other concurrently associated injury, making this case very rare. Even if the patient in our case had experienced any indirect trauma, indirect trauma causing the fracture to the coronoid process is unlikely. This is due to the fact that the coronoid process has no direct relation with any of the other cranial bones [4].

There are some theories that link the chronic use of a proton pump inhibitor to an increased risk of fractures and/or decreased bone mineral density. This could be due to the fact that insoluble calcium requires an acidic environment for optimal absorption. Proton pump inhibitors like omeprazole decrease the amount of gastric acid produced, thus increasing the pH of the gastric environment and potentially decreasing calcium absorption and bone mineralization. In the literature, there are some studies that link the use of omeprazole to a potential decrease in bone mineral density and/or an increased risk of fractures [6]. However, these studies mainly examined the risk of hip, spine, and wrist fractures in post-menopausal women taking a proton-pump inhibitor, and even then, causality between these factors has not been fully established. Facial fractures due to the risk of proton pump inhibitor use have not been widely studied, thus it is uncertain in our case that the patient experienced these bilateral coronoid process fractures due to the use of omeprazole.

Additionally, there is likely to be some association between the patient’s past history of TMJ problems and the current fractures. The first step to explore this association would be to review the full history to determine what kind of TMJ conditions she may have suffered from in the past. It is unlikely that she is experiencing a very rare condition called coronoid process hyperplasia because the height of the coronoid process doesn’t exceed the height of the condylar head on imaging to suggest coronoid hyperplasia [7]. This condition is characterized by excessive coronoid process growth, where mandibular movements become limited by the impaction of the coronoid process on the posterior portion of the mandible. This condition may occur in around five percent of patients with limited mouth-opening ability, with the coronoid process hyperplasia due to congenital coronoid hyperplasia or secondary to long-standing disk displacement without reduction [8]. Although this patient does not have a significant limitation in her incisal mouth opening [9], due to her reported long-standing history of TMJ problems and evidence of anterior displacement of the left TMJ on the most recent MRI, coronoid process hyperplasia should not be totally ruled out as a potential contributing factor. However, there are no reports found in the literature directly linking coronoid process hyperplasia with coronoid process fractures as of yet. Additionally, questions concerning sleep habits and positioning and symptoms of nighttime bruxism (jaw soreness or a headache in the morning) should be explored, as these are linked to chronic TMJ disorders.

Finally, there is an association between chronic TMJ disorders and mood disorders or other psychiatric comorbidities. This patient has a history of depression and mood disorder symptoms for which she was previously on treatment with Zoloft. Although it has been studied that mood disorders are associated with chronic TMJ pain, no association with coronoid process fractures has been reported [10].

## Conclusions

Given that there was no history of severe direct trauma, the most common cause of coronoid process fractures, the etiology of isolated fractures in this patient is idiopathic or unknown at this time. Albeit very rare, coronoid process hyperplasia should be given more attention especially in patients with chronic TMJ disorders and limited mouth opening, as it may be more common than previously thought. Exploring more into the patient’s history of previous TMJ disorders may help to uncover a definite etiology of her fractures. Treatment of this patient’s fractures through ORIF as described above may give this patient the most clinical benefit.
